# The Transcription Factor c-Jun Protects against Liver Damage following Activated β-Catenin Signaling

**DOI:** 10.1371/journal.pone.0040638

**Published:** 2012-07-06

**Authors:** Claudia Trierweiler, Hubert E. Blum, Peter Hasselblatt

**Affiliations:** 1 Department of Medicine II, University Hospital Freiburg, Freiburg, Germany; 2 Faculty of Biology, Freiburg University, Freiburg, Germany; University of Sherbrooke, Canada

## Abstract

**Background:**

The Wnt/β-Catenin signaling pathway is central for liver functions and frequently deregulated in hepatocellular carcinoma (HCC). Analysis of the early phenotypes and molecular events following β-Catenin activation is therefore essential for better understanding HCC pathogenesis. The AP-1 transcription factor c-Jun is a putative β-Catenin target gene and promotes hepatocyte survival, proliferation, and liver tumorigenesis, suggesting that c-Jun may be a key target of β-Catenin signaling in the liver.

**Methodology/Principal Findings:**

To address this issue, the immediate hepatic phenotypes following deletion of the tumor suppressor *Apc* and subsequent β-Catenin activation were analyzed in mice. The contribution of c-Jun to these phenotypes was dissected in double mutant animals lacking both, *Apc* and *c-Jun*. β-Catenin was rapidly activated in virtually all *Apc* mutant hepatocytes while c-Jun was induced only after several days, suggesting that its expression was rather a secondary event following *Apc* deletion in the liver. Loss of *Apc* resulted in increased hepatocyte proliferation, hepatomegaly, deregulated protein metabolism, and premature death. Interestingly, additional deletion of *c-Jun* did not affect hepatocyte proliferation but resulted in increased liver damage and mortality. This phenotype correlated with impaired expression of hepatoprotective genes such as Birc5, Egfr Igf1 and subsequently deregulated Akt signaling.

**Conclusions/Significance:**

These data indicate that c-Jun is not a primary target of β-Catenin signaling in the liver, but rather protects against liver damage, which in turn may promote liver tumorigenesis.

## Introduction

The Wnt/β-Catenin signal transduction cascade is a major regulator of liver development and hepatocyte function [1]. Moreover, β-Catenin is an established oncogene and defines a genetically distinct subset of hepatocellular carcinoma (HCC) [2]. Its activity therefore needs to be tightly controlled. Under resting conditions, β-Catenin is bound by a multiprotein complex consisting of Adenomatosis polyposis coli (Apc), Axin and Glycogen synthase kinase 3β and is targeted for proteasome-dependent degradation. Disruption of this complex, e.g., upon binding of Wnt ligands to their respective receptors or inactivating mutations of *Apc* results in stabilization and nuclear translocation of β-Catenin, where it cooperates with ternary complex factors (Tcf) to form a ternary complex with DNA responsive elements, thereby regulating expression of its target genes [3].

Genetic mouse models have been particularly useful to determine the functions of Apc and β-Catenin in the liver. Constitutive loss of *Apc* or gain of β-Catenin function results in early lethality [1], [4], [5]. In contrast, conditional and liver-specific knockout of *Apc* after birth results in increased hepatocyte proliferation, hepatomegaly and premature mortality within days, possibly due to perturbations in protein and ammonia metabolism [6], [7]. In addition, *Apc* acts as tumor suppressor in the liver since its mosaic deletion in only a subset of hepatocytes is sufficient to cause HCC [6]. Therefore, a detailed analysis of the molecular pathways following β-Catenin activation is essential to better understand HCC pathogenesis with the potential to identify novel candidates for targeted therapies.

The immediate phenotypes of *Apc* loss are β-Catenin-dependent and rescued upon additional knockout of *Ctnnb1* (which encodes β-Catenin) [8]. *Apc* mutant mice are therefore valuable to study the functions of β-Catenin and its target genes in the liver [9]. Interestingly, it was previously shown that the phenotypes of *Apc* loss in the intestine, but not in the liver, are rescued by additional knockout of *c-Myc* [8], [10]. However, the β-Catenin-dependent signaling network in the liver is still incompletely understood.

Several lines of evidence suggest that the transcription factor c-Jun may be an important mediator of β-Catenin functions in the liver. c-Jun is a member of the AP-1 (activator protein 1) complex that contains dimers of either Jun (c-Jun, JunB and JunD), Fos (c-Fos, FosB, Fra-1 and Fra-2), Atf (activating transcription factor) or Maf (musculoaponeurotic fibrosarcoma) protein families [11]. c-Jun transcription is directly regulated by β-Catenin, at least in the intestine [12], [13]. Moreover, c-Jun is an important regulator of liver development and regulates hepatocyte proliferation after partial hepatectomy [14], [15], [16], [17]. c-Jun is also frequently expressed in human HCCs and acts as an oncogene in the liver [18], [19]. In addition, c-Jun promotes hepatocyte survival under stress conditions such as acute hepatitis and endoplasmic reticulum (ER) stress [20], [21]. We therefore addressed the functions of c-Jun in response to hepatic *Apc* loss using double mutant mice lacking both, *Apc* and *c-Jun*. Our findings indicate that c-Jun is not essential for hepatocyte proliferation in this setting, but rather protects against liver damage, presumably by interacting with growth factor signaling and subsequent activation of Akt .

## Results

### Hepatic loss of *Apc* results in rapid activation of β-Catenin but delayed induction of c-Jun expression

Compound mutant mice carrying conditional alleles of *Apc* and *c-Jun* as well as an inducible Cre recombinase under control of the interferon-responsive *Mx1* promoter were generated to analyze the functional impact of c-Jun on the immediate phenotypes of *Apc* loss in the liver. Mice were injected with double stranded RNA (poly(I·C)) in order to induce Cre-mediated recombination and to generate single *Apc* knockout (*SKO*) or *Apc*/*c-Jun* double knockout mice (*DKO*).

**Figure 1:**
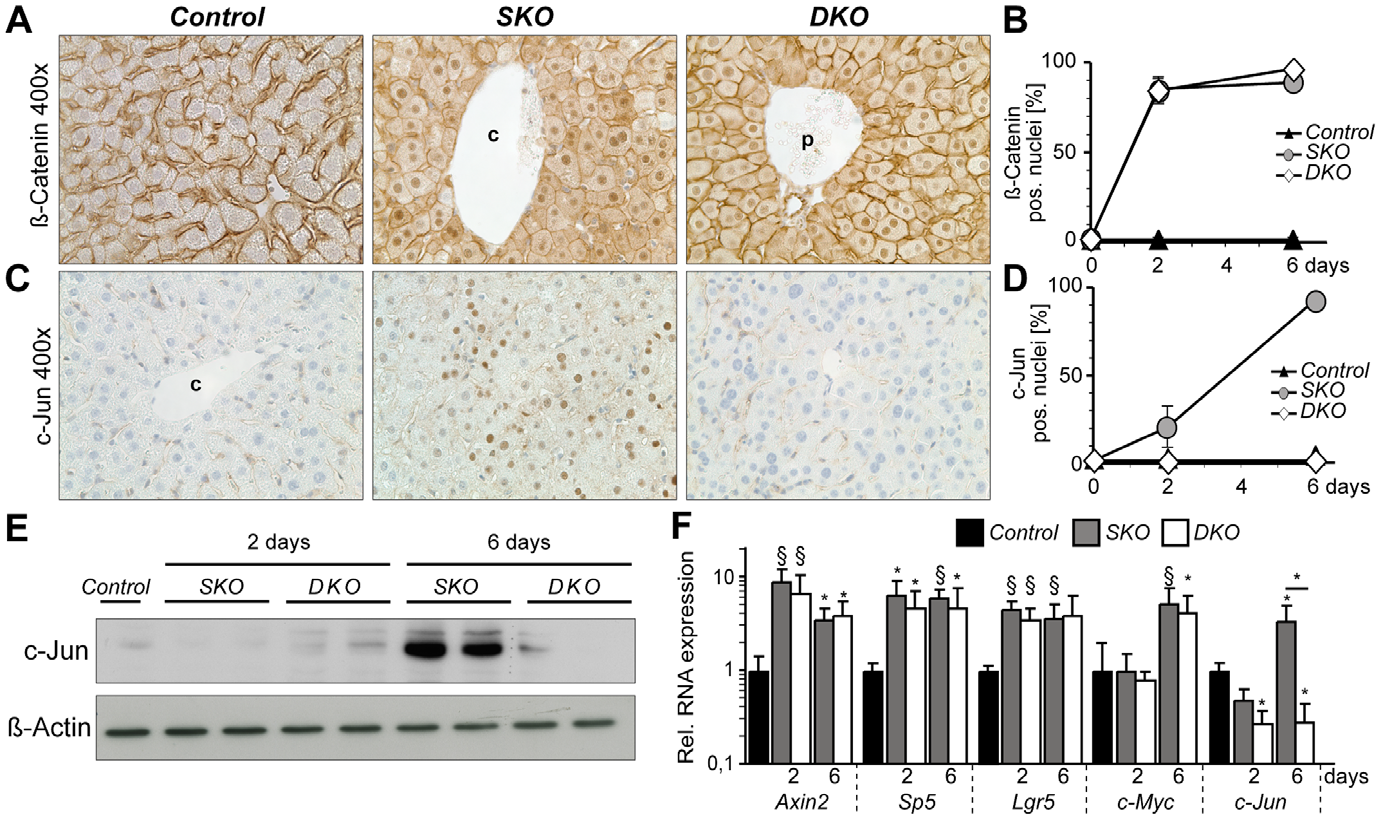
Hepatic loss of *Apc* results in activation of β-Catenin, but delayed induction of c-Jun. (**A–D**) Immunohistochemistry was performed to analyze the expression of β-Catenin (**A**) and c-Jun (**C**) in livers from mice with the indicated genotypes 6 days after Cre-mediated recombination. (**B, D**) Nuclear expression of β-Catenin and c-Jun in hepatocytes was quantified at the indicated time points and is given in % ± S.D. (*n* = 6–11 mice per genotype). (**E**) c-Jun expression of liver lysates from mice with the indicated genotypes and timepoints was analyzed by immunoblotting. β-Actin was used as loading control. (**F**) Hepatic expression of putative β-Catenin target genes at different timepoints following Cre induction was determined by qPCR and is given as relative expression compared to control livers (*n* = 4–5 livers/genotype); §, *P*≤0.01; *, *P*≤0.05 as compared to control livers.

Since deletion of *Apc* leads to stabilization and subsequent nuclear translocation of β-Catenin, immunohistochemistry for β-Catenin was performed to functionally assess the *Apc* recombination efficiency. β-Catenin was predominantly localized on cell membranes in control livers, but translocated to the nucleus in most hepatocytes in *SKO* and *DKO* mice as early as 2 days after poly(I·C) injection (Fig. 1A, B). c-Jun expression was almost absent in control livers. In *SKO* livers, nuclear c-Jun expression was detected by immunohistochemistry only in a small subset of hepatocytes 2 days after loss of *Apc* and further increased thereafter (Fig. 1C, D). Immunoblotting confirmed that c-Jun expression in *SKO* livers mainly occurred 6 days after Cre induction (Fig. 1E). In contrast, c-Jun expression was absent in *DKO* hepatocytes (Fig. 1.C–E) and efficient recombination was also evident by PCR analysis of the respective loci (data not shown). Quantitative PCR analysis was performed to analyze the expression kinetics of β-Catenin target genes in more detail. Nuclear translocation of β-Catenin in *SKO* and *DKO* livers at 2 days after Cre induction correlated with increased expression of established β-Catenin target genes such as Axin2, the transcription factor Sp5 and the intestinal stem cell marker Lgr5. In contrast, expression of c-Jun and c-Myc was delayed and only apparent after 6 days following Cre induction. This was also consistent with previous observations that c-Myc may not be a direct transcriptional target of β-Catenin in the liver [9], [22]. In conclusion, our findings indicate that *Mx1Cre* mediates efficient recombination of each, the *Apc* and *c-Jun* locus. Moreover, the delayed and more restricted expression pattern of c-Jun suggests that c-Jun is not a primary target of β-Catenin in the liver following loss of *Apc*.

### c-Jun is not required for β-Catenin-dependent hepatocyte proliferation and liver zonation

**Figure 2:**
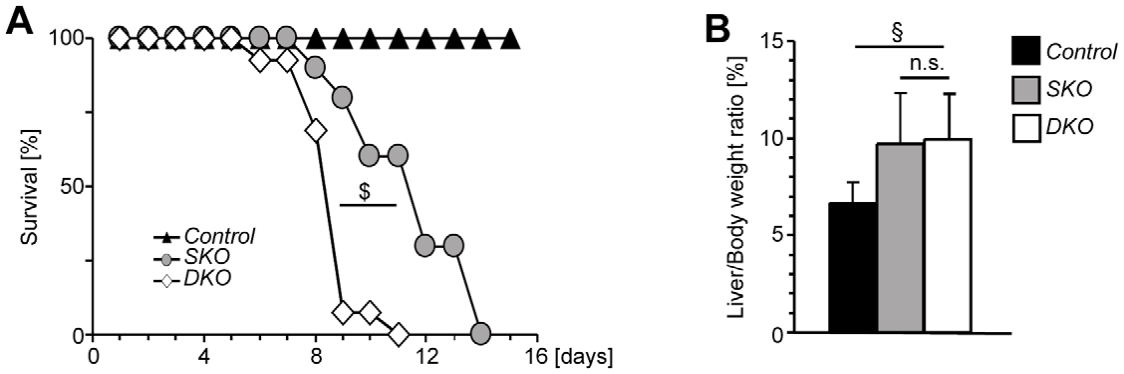
c-Jun prolongs mouse survival upon *Apc* **loss.** (**A**) Survival of mice with the indicated genotypes was analyzed for 15 days following induction of Cre and is shown as Kaplan Meier blot in [%]. (*n* = 10–16 mice per genotype; $, *P* = 0.0001). (**B**) Liver weight of mice with the indicated genotypes was determined prior to death and is given as liver/body weight ratio in [%]. (*n* = 10–16 mice/genotype; §, *P*<0.001; n.s., not significant).

**Figure 3:**
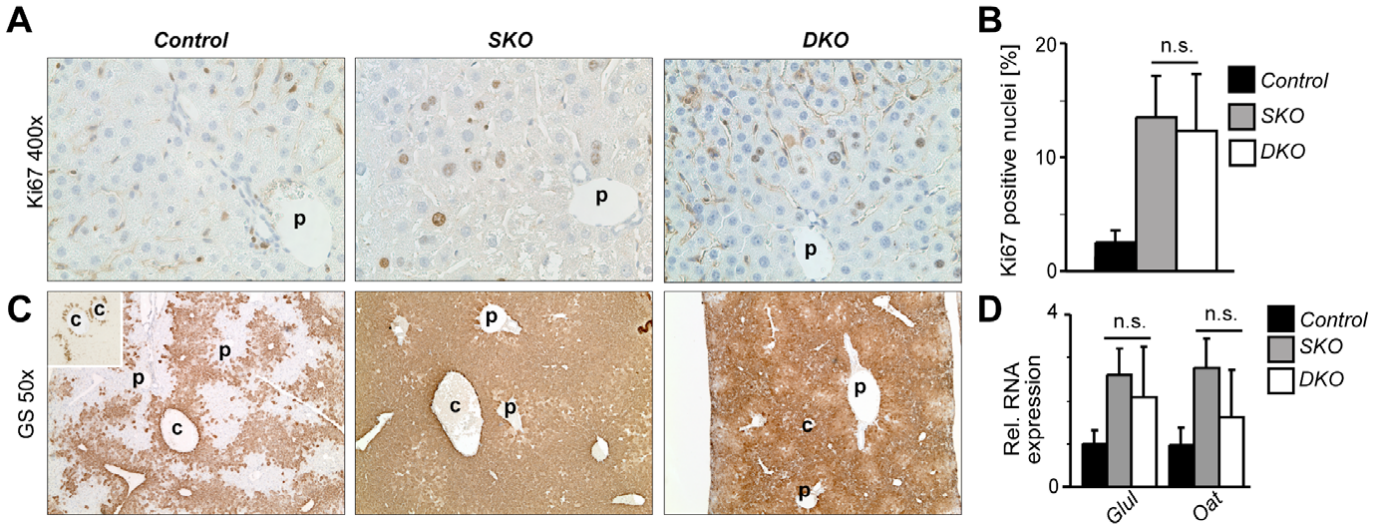
c-Jun is not essential for hepatocyte proliferation and liver zonation following *Apc* **loss.** (**A**) Hepatocyte proliferation was analyzed at 6 days following Cre induction by immunohistochemistry for the proliferation marker Ki67. (p, portal vein). (**B**) The percentage of Ki67 positive hepatocytes is shown in [%] ± S.D. (*n* = 6–11 mice/genotype; n.s., not significant). (**C**) Immunohistochemistry for the β-Catenin target gene and zonation marker glutamine synthetase (GS) was performed in livers with the indicated genotypes. p, portal vein; c, central vein. (**D**) Hepatic expression of the indicated genes at 6 days following Cre induction was determined by qPCR and is given as relative expression compared to control livers (*n* = 5 livers/genotype).

Loss of *Apc* in the liver results in increased hepatocyte proliferation, subsequent hepatomegaly and mortality within days [8]. Consistent with these findings, *SKO* animals died after a median of 11 days following poly(I·C) injection. Interestingly, median survival was significantly reduced to 8 days in *DKO* animals, while survival of control animals was not affected (Fig. 2A). Moderate hepatomegaly was evident in all *Apc* mutants irrespective of c-Jun expression, indicating that this phenotype was independent of c-Jun (Fig. 2B). Hepatocyte proliferation was determined by immunohistochemistry for the proliferation marker Ki67. Interestingly, the hepatocyte proliferation index increased to the same extent in *SKO* and *DKO* livers (Fig. 3A, B), indicating that c-Jun is not an essential mediator of β-Catenin-dependent hepatocyte proliferation.

Apc and β-Catenin also regulate the spatial expression pattern of genes involved in hepatic glutamine and ammonia metabolism thereby controlling a process called liver zonation, which is profoundly disturbed upon loss of *Apc* [7] [23]. However, expression of the established β-Catenin target gene and zonation marker glutamine synthetase (GS, encoded by the *Glul* gene), as well as ornithine aminotransferase (*Oat*) was comparable in *SKO* and *DKO* livers as determined by qPCR (Fig. 3D). Immunohistochemistry further revealed that GS expression was confined to pericentral hepatocytes in *Apc* ^f/f^ control animals (Fig. 3C), although it should be noted that GS staining is more restricted to the most pericentral hepatocyte layer in *Apc* ^+/+^ livers (small inset in Fig. 3C), consistent with the previous finding that conditional *Apc* alleles are hypomorphic [24]. In contrast, GS was similarly expressed throughout the liver in *SKO* and *DKO* mutants, suggesting that c-Jun is not essential for regulating liver zonation.

### c-Jun attenuates liver damage following *Apc* loss

**Figure 4:**
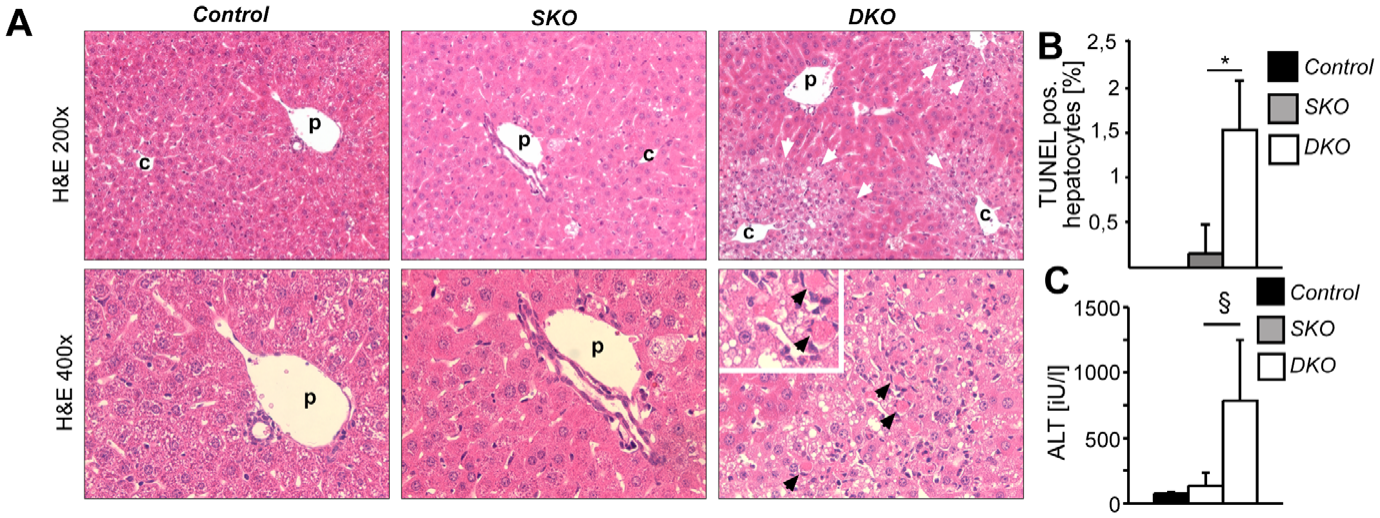
c-Jun attenuates liver damage following *Apc* **loss.** (**A**) Liver histology of mice with the indicated genotypes was analyzed 6 days after Cre induction. Pronounced hypertrophy of periportal hepatocytes was evident in *DKO* livers. By comparison, hepatocytes in the pericentral compartment (white arrows) were smaller and contained many vacuoles and scattered eosinophilic apoptotic bodies (black arrowheads; p, portal vein; c, central vein). (**B**) Apoptosis was analyzed by TUNEL assay, quantified and the hepatocyte apoptosis index is given in [%] ± S.D., (*n* = 3–4 livers/genotype). (**C**) Liver damage in mice with the indicated genotypes was determined by analysis of serum ALT ± S.D. 6 days after Cre induction (*n* = 5–11 mice/genotype; §, *P*<0.001).

c-Jun is a stress-responsive immediate early gene and its delayed expression may reflect increased cell stress occurring at later timepoints following *Apc* loss. Histological analyses revealed that deletion of *Apc* resulted in moderate hypertrophy of periportal hepatocytes in *SKO* mice, which was more pronounced in *DKO* livers. In contrast, hepatocytes within the pericentral compartment of *DKO* livers were considerably smaller and frequently contained vacuoles, which may reflect increased hepatic stress (Fig. 4A, white arrows). Moreover, scattered apoptotic hepatocytes were evident by H&E staining (Fig. 4A, black arrowheads at higher magnification in the lower panel). Although hepatocyte apoptosis as determined by TUNEL assay was significantly induced in *DKO* livers, it was a rather rare event (Fig. 4B). However, serum transaminase concentrations were strongly increased in *DKO* animals, reflecting increased liver damage following *Apc* loss (Fig. 4C). Interestingly, increased liver damage was also observed in *Apc* mutant mice heterozygous for *c-Jun*, in which c-Jun expression was reduced by 60% as determined by immunohistochemistry (data not shown). These findings indicate that c-Jun protects against liver damage following loss of *Apc* and that these functions are likely affected by *c-Jun* gene dosage.

### Loss of *c-Jun* affects the expression of genes related to growth factor signaling and Akt phosphorylation

**Figure 5:**
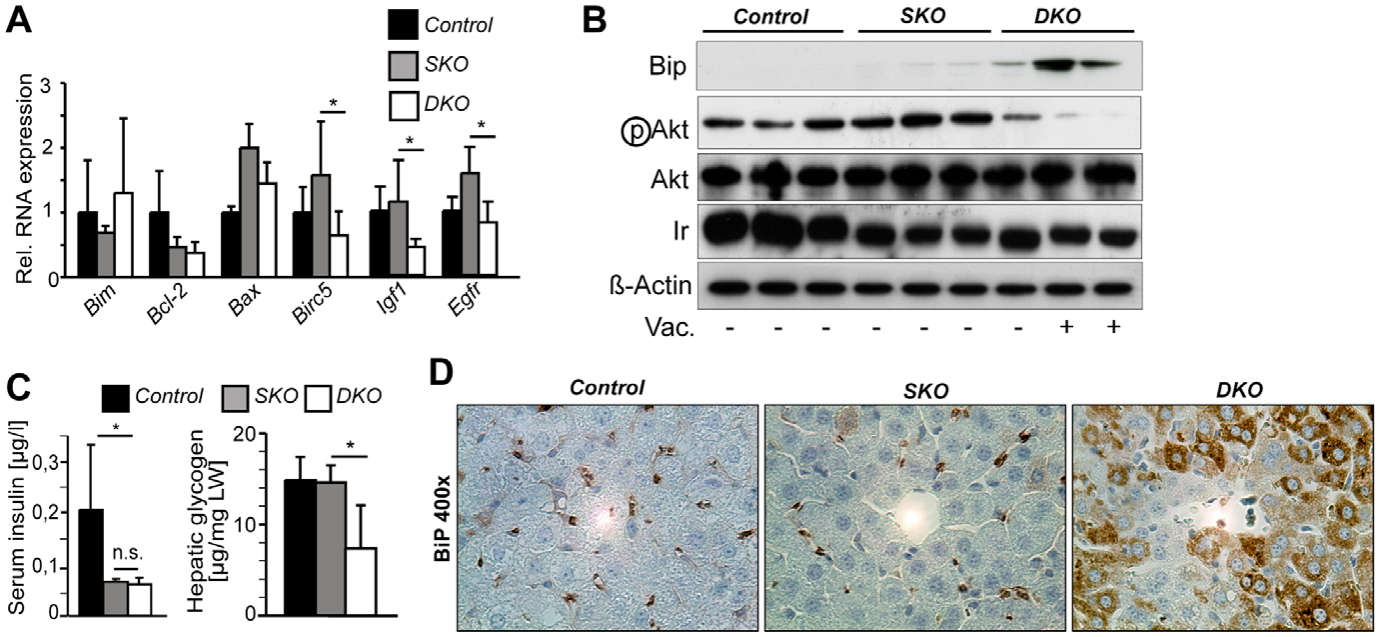
Loss of *c-Jun* affects the expression of genes related to growth factor signaling and Akt. (**A**) Hepatic expression of the indicated genes at 6 days upon Cre induction was determined by qPCR and is given as relative expression compared to control livers (*n* = 5 livers/genotype); *, *P*≤0.05. (**B**) Expression of the indicated proteins in total liver lysates of mice with the indicated genotypes was determined by immunoblotting. Ir, Insulin receptor. β-Actin was used as control. Samples from livers with strongly increased hepatocyte vacuolization are indicated. (**C**) Hepatic glycogen content and serum insulin concentrations were analyzed 6 days after Cre induction (n = 4–11/genotype); *, *P*≤0.05. (**D**) BiP expression predominantly occurred in vacuolated hepatocytes in DKO mice as determined by immunohistochemistry.

Expression analysis of candidate genes related to cell survival revealed that expression of the hepatoprotective genes Birc5 (also known as Survivin), insulin like growth factor 1 (Igf1) and epidermal growth factor receptor (Egfr) was reduced in *DKO* livers, while expression of the apoptosis-related BH3 genes Bim, Bax and Bcl-2 was not affected (Fig. 5A). Importantly, Igf1 and Egfr expression was also reduced in livers lacking *c-Jun* specifically in hepatocytes (Figure S1). Igf1 and other signaling cascades such as the insulin pathway converge at the serine/threonine kinase Akt, a central regulator of cell survival and metabolism. Importantly, Akt phosphorylation was strongly reduced in *DKO* livers (Fig. 5B). Reduced Akt phosphorylation in *DKO* livers was likely not mediated by insulin, since serum insulin concentrations and hepatic insulin receptor expression were reduced to the same extent in *SKO* and *DKO* mice (Fig. 5B,C). Increased hepatocyte vacuolization observed in *DKO* livers resembled the phenotype of sustained chemically-induced ER stress in livers lacking *c-Jun* [21]. In keeping with this notion, expression of the chaperone and ER stress marker BiP was strongly induced in *DKO* livers and predominantly occurred in vacuolated hepatocytes (Fig. 5B,D). However, expression of other ER stress marker genes including *Gadd153*, *Gadd34* and *XBP-1* splicing was not altered in *DKO* as compared to *SKO* livers (data not shown). However, BiP (also known as glucose regulated protein 78, Grp78) expression also occurs during other stress responses such as starvation. In keeping with this notion, hepatic glycogen content was specifically reduced in *DKO* livers (Fig. 5C). These findings suggest that the composite loss of *Apc* and *c-Jun* results in deregulated signaling through growth factors and Akt, which in turn may exacerbate liver damage in the absence of *c-Jun*.

## Discussion

Although Apc may exert functions independent of β-Catenin [25], *Apc* mutant mice are an established model to study the immediate liver phenotypes and changes in gene expression following β-Catenin activation [4], [7], [8], [9]. Here, we studied the impact of the AP-1 transcription factor c-Jun on these phenotypes using compound mutant mice lacking both *Apc* and *c-Jun*.

A key finding of our study is the observation that c-Jun is likely not a direct β-Catenin target gene and not an essential mediator of the primary phenotypes of *Apc* loss in the liver such as deregulated liver zonation, hepatocyte proliferation and subsequent hepatomegaly. c-Jun has previously been identified as a β-Catenin target gene in the intestine and mediates β-Catenin-dependent intestinal tumorigenesis in *Apc* ^Min^ mice [12], [13]. However, these functions are likely dependent on the experimental system since c-Jun expression was also not required for proliferation of small intestinal epithelial cells upon knockout of *Apc* or an activating mutation of *Ctnnb1* as well as during colitis-associated colorectal cancer [26].

By contrast, hepatic loss of *Apc* and *c-Jun* rather resulted in increased liver damage and mortality. It should be noted that these phenotypes may also be influenced by extrahepatic functions of c-Jun, since *Mx1Cre*-mediated recombination also occurred in extrahepatic tissues such as bone marrow and spleen. It is therefore uncertain whether the increased mortality of *DKO* mice can be attributed to increased liver damage alone and the extrahepatic functions of c-Jun and causes of death following *Apc* loss need to be analyzed in more detail in the future. Moreover, it has been shown previously that *Mx1Cre*-mediated deletion of *IKKβ* in non-parenchymal liver cells and hepatocytes results in opposite tumorigenesis phenotypes as compared to hepatocyte-specific deletion alone [27]. However, comparable results have not been reported upon *Mx1Cre*-mediated deletion of *c-Jun*, at least during chemically induced liver carcinogenesis and acute T-cell-mediated hepatitis [18], [20]. On the other hand, inducible recombination of *Apc* and *c-Jun* was highly efficient by using *Mx1Cre* and the hepatic phenotypes observed in these mice closely resembled previous reports of hepatic *Apc* loss by using different recombination approaches [6], [8]. Our mouse model can therefore be considered to correctly recapitulate the phenotypic hallmarks of hepatic *Apc* loss.

The hepatoprotective functions of c-Jun observed here are consistent with the emerging concept that c-Jun promotes hepatocyte survival during diverse stress conditions such as acute hepatitis, hepatic ER stress responses and liver tumorigenesis [18], [20], [21]. On the molecular level, increased liver damage in *DKO* livers correlated with impaired expression of genes implicated in the regulation of hepatocyte survival such as Birc5 and Egfr, which have both been reported as putative direct c-Jun and β-Catenin target genes in other experimental systems [8], [28], [29], [30]. Moreover, composite loss of *Apc* and *c-Jun* resulted in impaired Igf1 expression, which also occurred in livers specifically lacking *c-Jun* in hepatocytes, suggesting that Igf1 expression is indeed regulated by c-Jun in hepatocytes.

Nutrient availability, insulin and related growth factors regulate cell survival by activation of the serine/threonine kinase Akt, which is a master regulator of cell metabolism, survival, growth and cancer and frequently activated in HCC [31], [32], [33]. Moreover, Akt substantially contributes to regeneration and tumorigenesis following loss of *Apc* in the intestine [34]. Akt phosphorylation was substantially reduced in *DKO* livers, suggesting that c-Jun may directly or indirectly interact with hepatoprotective Akt/mTor signaling in this context. Impaired Akt phosphorylation also correlated with reduced hepatic glycogen content and it is therefore tempting to speculate that c-Jun may be involved in the regulation of metabolic responses in the liver. In addition, Birc5, Egfr, Igf as well as downstream Akt signaling all promote liver tumorigenesis [32], [35], suggesting that c-Jun may promote the formation of β-Catenin-dependent HCC by directly or indirectly regulating the expression of these genes.

In conclusion, we provide evidence that c-Jun is not a primary target of β-Catenin signaling in the liver, but rather protects against liver damage in this context. These findings provide a rationale to further explore the interactions of c-Jun with hepatoprotective Akt signaling in metabolic liver disease, hepatocarcinogenesis and tumor cell metabolism.

## Materials and Methods

### Animals

Mice with conditional alleles for *c-Jun* or *Apc* (c-*Jun* ^f/f^ and *Apc^f^* ^/f^, respectively) were crossed with transgenic mice expressing Cre under the control of the interferon-responsive *Mx1* promoter [16], [36], [37]. Tg(*Mx1Cre) Apc* ^f/f^ *c-Jun* ^+/+^ and Tg(*Mx1Cre) Apc* ^f/f^ *c-Jun* ^f/f^ animals were used to generate single (*SKO*) and double knockout mice (*DKO*), respectively. Cre-mediated recombination was induced by injection of polyinosine-polycytidylic acid (poly (I·C), 15 µg/g BW, Amersham Biosciences, Piscataway, NJ) and mice were analyzed at the indicated time points. Mice were maintained on a mixed genetic background (C57BL/6×129/Sv) and housed under specific-pathogen free conditions. Littermates not expressing Cre were used as controls. Floxed *Apc* alleles are considered to have hypomorphic functions [24]. Pilot experiments comparing *Apc* ^f/f^ c*-Jun* ^f/f^ with *Apc* ^+/+^ c*-Jun* ^f/f^ mice revealed no differences in liver size, hepatocyte proliferation or RNA expression of the genes studied here, indicating that the phenotypes of the controls used here was not substantially affected by the floxed *Apc* alleles (Figure S2). For some control experiments, mice lacking *c-Jun* specifically in hepatocytes (*Tg(AlfpCre) c-Jun* ^f/f^, *c-Jun* ^Δli^) were used [11]. All animals received humane care and experiments were performed in accordance with local and institutional regulations. All experiments for this study were specifically approved by the local animal ethics committee (Regierungspräsidium Freiburg, Germany, permit number G08/12).

### Histology and immunohistochemistry

For histology, livers were fixed in 3.7% neutral buffered formaldehyde at 4°C overnight and embedded in paraffin. Immunohistochemistry was performed using the Envision kit (DAKO, Hamburg, Germany) and antibodies for c-Jun (#9165; Cell Signaling, NEB, Frankfurt, Germany), β-Catenin (#610154; BD Biosciences, San Diego, California), Glutamine synthetase (#610518; BD Biosciences), Ki67 (#301119; Novocastra; Newcastle, UK) and BiP (#3177, Cell Signaling). Slides were counterstained with Hematoxylin (Sigma, Schnelldorf, Germany). H&E stainings were performed according to standard protocols. Microscopy was performed on a Zeiss Observer Z1 with SPOT Imaging software. The percentage of hepatocytes expressing c-Jun, β-Catenin or Ki67 was determined by counting hepatocyte nuclei within 3 random 400× magnification fields per mouse (n ≥ 6 livers per genotype). TUNEL assays were performed using the *in situ* cell death detection kit (Roche, Mannheim, Germany).

### Cytotoxicity assays and liver glycogen analysis

Hepatocyte damage was determined by the level of serum transaminases and serum glucose concentrations were analyzed using semi-automated clinical routine methods. Liver glycogen concentrations were analyzed using a glycogen assay kit (BioCat, Heidelberg, Germany) according to the manufacturer's protocol.

### Western blot analysis and ELISA

Total liver lysates were analyzed by immunoblot using antibodies for β-Actin (Sigma), insulin receptor (sc711, Santa Cruz), BiP (#3177, Cell Signaling), p-Akt (Ser473 #4060, Cell Signaling) and Akt (# 4685, Cell Signaling). Serum insulin concentrations were determined using the insulin (rat) ultrasensitive ELISA kit with 75% mouse sensitivity (EIA-2943; DRG Instruments, Marburg, Germany).

### qPCR

Upon isolation of total liver RNA using Qiazol (Qiagen, Hilden, Germany), cDNA synthesis was performed using the first strand cDNA synthesis kit (Fermentas, St. Leon-Rot, Germany). qPCR was performed with SYBR Green (Invitrogen, Karlsruhe, Germany) on a 480 Lightcycler (Roche) and the expression levels of transcripts were calculated with the comparative CT method. The individual RNA levels were normalized for *Hprt*. Primer sequences are available upon request.

### Statistics

Data in bar graphs represent mean ± S.D. as indicated. Statistical analysis was performed using the nondirectional two-tailed Student's *t* test. Statistical analysis of mouse survival was determined by log rank test.

## Supporting Information

Figure S1 **Expression of the indicated genes was analyzed in control livers and livers from mice specifically lacking** ***c-Jun*** **in hepatocytes (** ***c-Jun*** **^Δli^). (** ***n*** = 3 livers/genotype); *, ***P*** **≤0.05; §,** ***P*** **≤0.01.**
(TIF)

Figure S2 **Hypomorphic floxed** ***Apc*** **alleles do not affect liver size, hepatocyte proliferation and hepatic gene expression.** (**A**) Liver weight of mice with the indicated genotypes was determined and is given as liver/body weight ratio in [%]. (*n*>4 mice/genotype). (**B**) The percentage of Ki67 positive hepatocytes is shown in [%] (*n*>4 livers/genotype). (**C**) Hepatic expression of the indicated genes was determined by qPCR and is given as relative expression compared to control livers (*n*>3 livers/genotype).
(TIF)
