# Supplementary figures and images for: The Transcription Factor c-Jun Protects against Liver Damage following Activated β-Catenin Signaling
Source: PLoS One. 2012 Jul 6;7(7):e40638. doi: 10.1371/journal.pone.0040638 (PMC3391288; doi:10.1371/journal.pone.0040638)

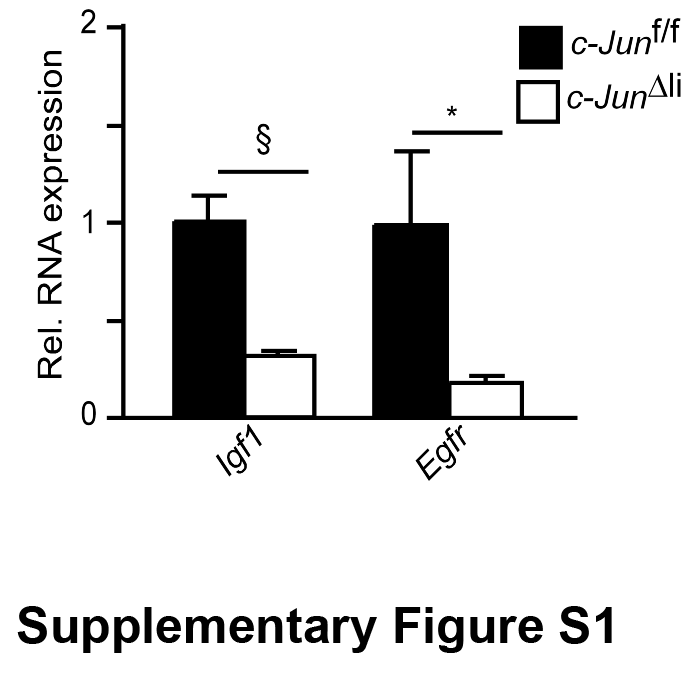

Supplement: Figure S1 — Expression of the indicated genes was analyzed in control livers and livers from mice specifically lacking c-Jun in hepatocytes ( c-Jun Δli). ( n = 3 livers/genotype); *, P ≤0.05; §, P ≤0.01. (TIF) [file pone.0040638.s001.tif]

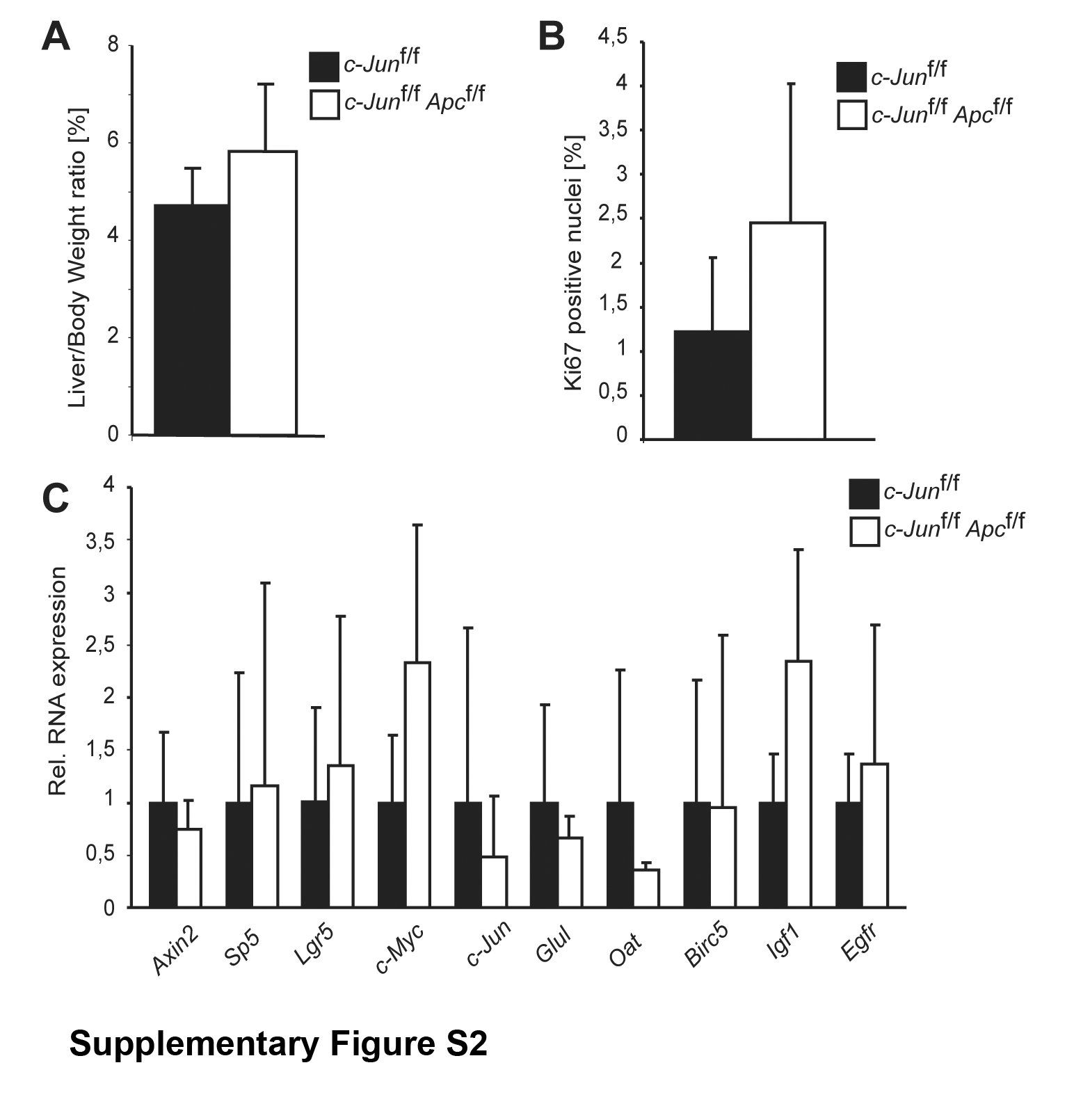

Supplement: Figure S2 — Hypomorphic floxed Apc alleles do not affect liver size, hepatocyte proliferation and hepatic gene expression. (A) Liver weight of mice with the indicated genotypes was determined and is given as liver/body weight ratio in [%]. (n>4 mice/genotype). (B) The percentage of Ki67 positive hepatocytes is shown in [%] (n>4 livers/genotype). (C) Hepatic expression of the indicated genes was determined by qPCR and is given as relative expression compared to control livers (n>3 livers/genotype). (TIF) [file pone.0040638.s002.tif]
